# Expression Profiling and Characterization of *HcTGA5* Gene in Kenaf Under Hormonal Treatments and Its Association with Resistance to Root-Knot Nematode

**DOI:** 10.3390/ijms27188249

**Published:** 2026-09-16

**Authors:** Jiguang Luo, Xuewu Wang, Xiangping Zeng, Zhixiang Zhao, Miancai Chen, Meiying Fu, Huifang Wang

**Affiliations:** Key Laboratory of Plant Diseases and Pests of Hainan Province, Institute of Plant Protection (Research Center of Quality Safety and Standards for Agro-Products, Hainan Academy of Agricultural Sciences), Hainan Academy of Agricultural Sciences, Haikou 571100, China; luojiguang@hnaas.org.cn (J.L.); laserluo@163.com (X.W.); zengxiangping@hnaas.org.cn (X.Z.); zhaozhixiang@hnaas.org.cn (Z.Z.); miancaichen@163.com (M.C.); fumeiying@hnaas.org.cn (M.F.)

**Keywords:** *Hibiscus cannabinus*, *Meloidogyne enterolobii*, *HcTGA5*, disease-resistant plants

## Abstract

Kenaf (*Hibiscus cannabinus* L.), a member of the Malvaceae family, has gained increasing research interest owing to its therapeutic attributes. Among pathogens affecting tropical kenaf cultivation, *Meloidogyne enterolobii* is the most economically destructive disease, causing severe yield loss and quality deterioration. Consequently, the identification of *M. enterolobii*-resistant kenaf germplasms and the elucidation of the underlying molecular pathways are primary objectives for plant breeders. In this study, the gene encoding a *TGA*-like protein associated with root-knot nematode resistance was cloned from kenaf via reverse transcription polymerase chain reaction (RT-PCR) and the rapid amplification of cDNA ends (RACE), and designated as *HcTGA5* gene. The full-length cDNA is 798 bp, encoding a 230-amino acid protein (GenBank accession: PZ944973). The application of phytohormones (jasmonic acid, JA; salicylic acid, SA; ethylene, ET) profoundly affected *HcTGA5* gene expression with specific temporal patterns: peak expression was observed at 6 h after ET treatment, followed by 48 h after SA, and 24 h after JA treatment. *HcTGA5* gene demonstrated an ‘initial upregulation followed by decline’ trend in response to ET treatment, whereas SA and JA triggered a ‘rise–fall–rise’ fluctuation. The data demonstrate the temporal modulation of *HcTGA5* gene expression by phytohormones, suggesting its function in hormone-mediated defense signaling. A comparative analysis revealed markedly elevated *HcTGA5* gene expression in *M. enterolobii*-resistant germplasms compared to susceptible ones, indicating its possible association with nematode resistance. In conclusion, *HcTGA5* gene may play an integral role in kenaf’s defense against *M. enterolobii*. The study establishes a basis for investigating the nematode resistance mechanisms of kenaf and gives a theoretical framework for the genetic enhancement and disease management of kenaf.

## 1. Introduction

The immune response and adaptive mechanisms of plants to nematode infection are sophisticated [1,2]. The plant initiates a series of signaling cascades to combat nematode infestation [3,4]. Kenaf (*Hibiscus cannabinus* L.) is an annual herbaceous fiber crop and the largest mallow fiber plant worldwide, classified within the genus *Hibiscus* [5]. Kenaf is a diploid species (2n = 36) that requires short days and is an annual herb used for twine fiber production, as well as for the development of adsorbents, high-biomass ropes, textiles, fibers in recycled plastics, and livestock feed [6,7]. In comparison to other cannabis varieties, kenaf exhibits a broad geographical distribution owing to its robust ecological tolerance to several soil types [8].

Kenaf originated in Africa and has been cultivated for over than 4000 years. China introduced the Indian variety “Madras Red” in 1908 [9]. Kenaf is predominantly grown for the extraction of its bast fiber. It exhibits several significant biological traits, including rapid growth (with a biological yield 3–4 times greater than that of trees), elevated carbon dioxide absorption capacity (4–5 times that of trees), robust stress resistance (to drought, saline–alkali conditions, and nutrient-poor soils), extensive adaptability, and a substantial yield of fast-growing fibers. Kenaf is predominantly cultivated in the provinces of Henan, Anhui, Zhejiang, Fujian, Hunan, Guangxi, Guangdong, and Hainan, among others, in China [10,11,12]. In recent years, the cultivation area of kenaf has markedly diminished due to issues including minimal economic returns and the substantial degradation of water resources resulting from ineffective degumming methods. The production of kenaf fiber fails to satisfy the requirements of the textile processing sector. Since almost 80% of the raw materials are sourced from Bangladesh, the expenses are comparatively elevated. A limited number of plant disease resistance genes identified in current studies are directly involved in disease and stress resistance. However, the expression pathways of these genes that are directly involved in disease resistance are complex. Key regulatory elements in the upstream of plants regulate the activation and deactivation of resistance signaling pathways. When a plant encounters external stress, particular signaling molecules within the plant interact with receptor proteins in the cells, thereby activating the expression of relevant resistance genes by modulating various intracellular transcription factors.

In the 1980s, researchers found that mutations or deletions in the promoter region containing the core sequence TGACG greatly decreased the expression of GUS (ß-glucuronidase) driven by the promoter, via their investigation of cauliflower mosaic virus (CaMV). Furthermore, the active element can adapt to different biological and abiotic stressors [13,14,15]. Katagiri et al. (1989) analyzed a tobacco cDNA library with the TGACG sequence as a reference, identifying two potential *TGA* sequences; this gene was the first *TGA* gene cloned from plants [16]. *TGA* transcription factors are an important gene family in plants. They regulate pathogenesis-related genes and disease resistance in *Arabidopsis thaliana* via their interaction with non-expressor of PR genes 1 (*NPR1*) [17]. Matthews et al. (2014) demonstrated that overexpression of the *Arabidopsis* gene *AtTGA2* in transgenic soybean roots diminished the formation of cysts by the soybean cyst nematode (SCN: *Heterodera glycines*) to 38% of the levels observed in control roots [18]. Pascual et al. (2024) studied the role of two transcription factors (*TGA 1a* and *TGA 2.1*) in *Mi-1*-mediated tomato resistance to the root-knot nematode *Meloidogyne javanica* and confirmed that *TGA1a* plays a role in this mechanism [19]. Tian et al. (2024) studied the differential expression patterns of *CmTGA* genes in melon plants in response to various hormones like salicylic acid (SA), methyl jasmonate (MeJA), and ethylene (ET), as well as a fungal pathogen *Stagonosporopsis cucurbitacearum*, which induces gummy stem blight in melon. These findings shed light on the important roles of specific *CmTGA* genes in plant immunity [20].

This study investigates the cloning, isolation, and identification of resistance genes against root-knot nematodes in kenaf, specifically using the susceptible kenaf cultivar “4391” and resistant cultivar “ACC-NO-4114”. Following inoculation with *Meloidogyne enterolobii*, total RNA was extracted via TRIzol, leading to the identification of the *HcTGA5* gene associated with nematode resistance through transcriptomic sequencing. The gene was cloned, and the rapid amplification of cDNA ends (RACE) technique was employed to amplify its 3′ and 5′ ends, enabling full-length gene assembly. Bioinformatics tools were utilized to analyze gene characteristics. Additionally, kenaf plants were treated with jasmonic acid (JA), SA, and ET to assess the gene’s response to these compounds. Comparative expression analysis of *HcTGA5* gene in disease-resistant and susceptible kenaf informs potential genetic improvements for nematode management.

## 2. Results

### 2.1. Transcriptomic Analysis of Kenaf Resistance

**Figure 1:**
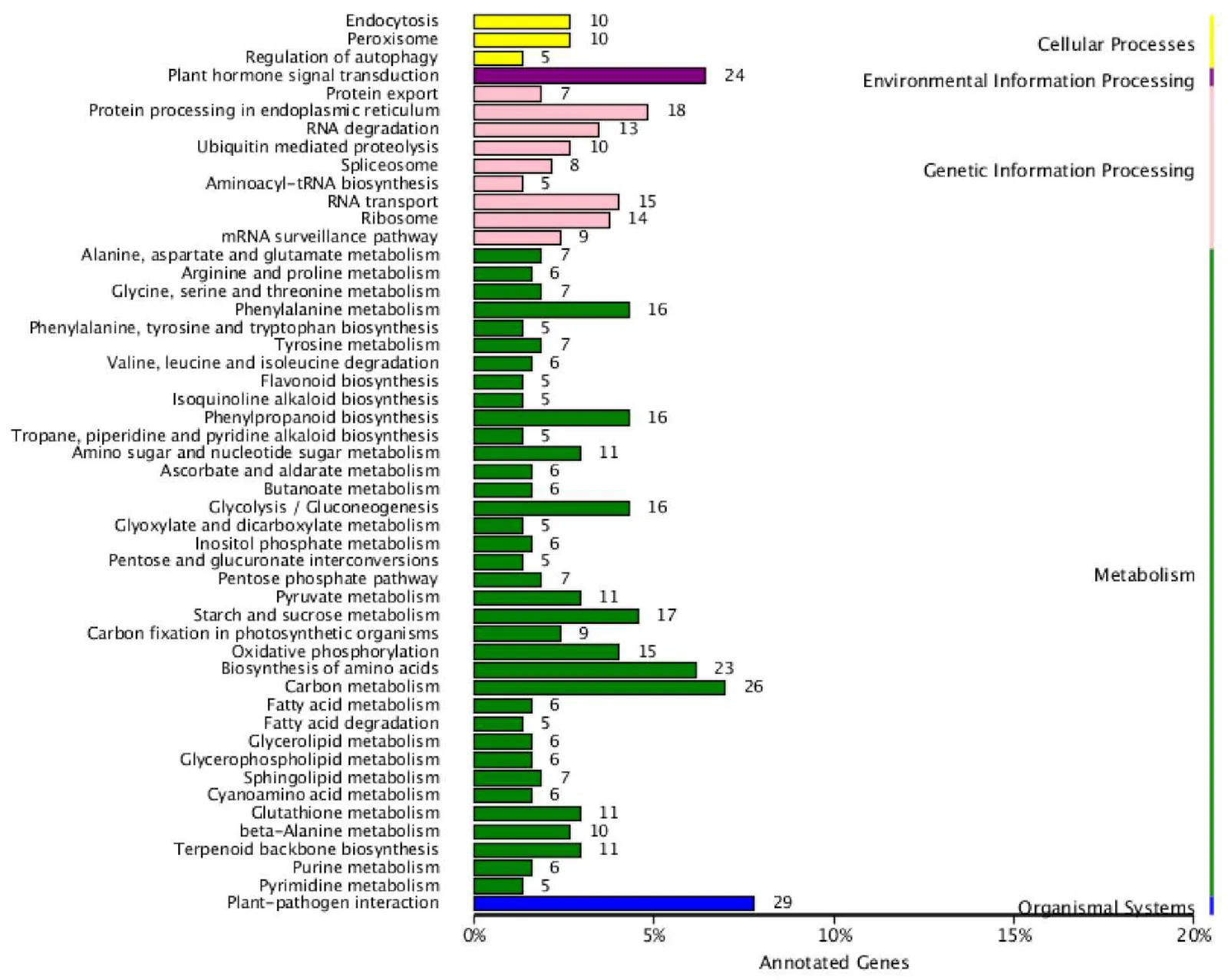
KEGG classification of differentially expressed genes. The ordinate is the name of the KEGG metabolic pathway, and the abscissa is the number of genes annotated to the pathway and their proportion to the total number of genes annotated.

**Figure 2:**
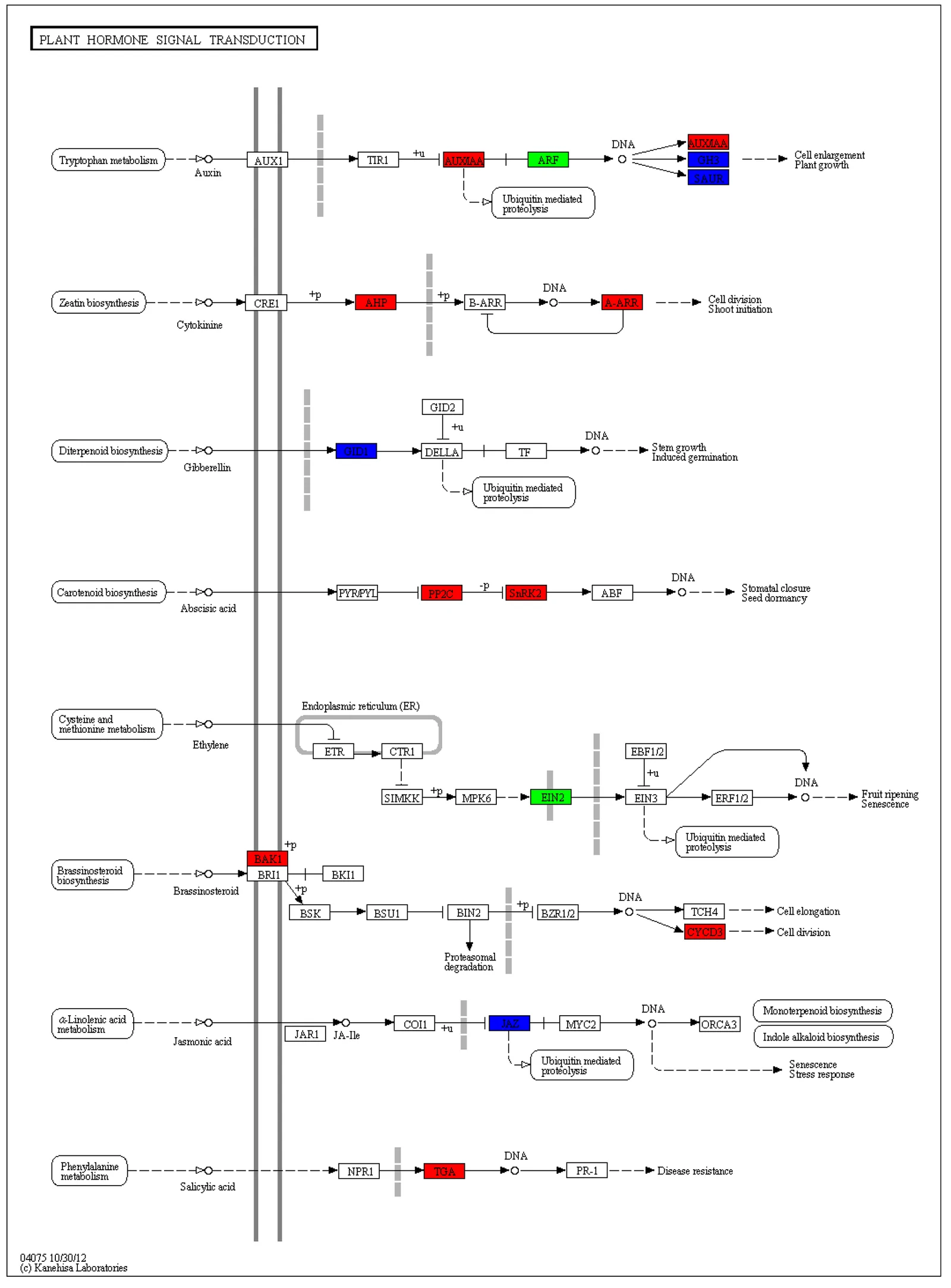
Schematic diagram of plant hormone signal transduction pathways for differentially expressed genes. Compared to the control group, the enzymes marked in the red box (*BAK1*, *TGA*, *CYCD3*, *PP2C*, *SnRK2*, *AHP*, *ARR*, *IAA*) are related to the up-regulated genes, and the enzymes marked in the green box (*ARF*, *EIN2*) are related to the down-regulated genes. Enzymes marked in blue boxes (*GH3*, *SAUR*, *GID1*, *JAZ*) are related to both up-regulated and down-regulated genes.

Total RNA was extracted from kenaf samples, and quality assessment revealed an OD260/OD280 ratio of 2.08, indicating high purity suitable for subsequent analyses. Simultaneously, two clear bands were visible (Figure S1), corroborating that the total RNA remained predominantly intact. These results validated the efficacy of the extraction procedure, which was subsequently used for further experiments. KEGG pathway enrichment analysis was performed based on the obtained differentially expressed genes (DEGs). The KEGG pathways were classified into five major categories: Metabolism, Genetic Information Processing, Environmental Information Processing, Cellular Processes, and Organismal Systems (Figure 1). The second category, Environmental Information Processing, included one enhanced pathway: Signal transduction of plant hormones. In this pathway, eight genes—*IAA*, *AHP*, *ARR-A*, *PP2C*, *SnRK2*, *BAK1*, *CYCD3*, and *TGA*—exhibited considerable up-regulation, whereas two genes, *ARF* and *EIN2*, demonstrated down-regulation (Figure 2). These hormonal signaling pathways have been acknowledged for their vital roles in plant–pathogen interactions.

Using transcriptomic data and previously established disease-resistance expression profiles, we screened for unigenes associated with resistance to *M. enterolobii* in kenaf. One of the annotated contigs was identified as corresponding to the *HcTGA5* gene. We designed gene-specific primers (detailed in Table S1) based on this contig sequence, and successfully cloned the full-length *HcTGA5* gene using the rapid amplification of cDNA ends (RACE) technique.

### 2.2. RACE at the 3′ End and RACE Amplification at the 5′ End of Root-Knot Nematode Resistance-Related Gene HcTGA5 in Kenaf

**Figure 3:**
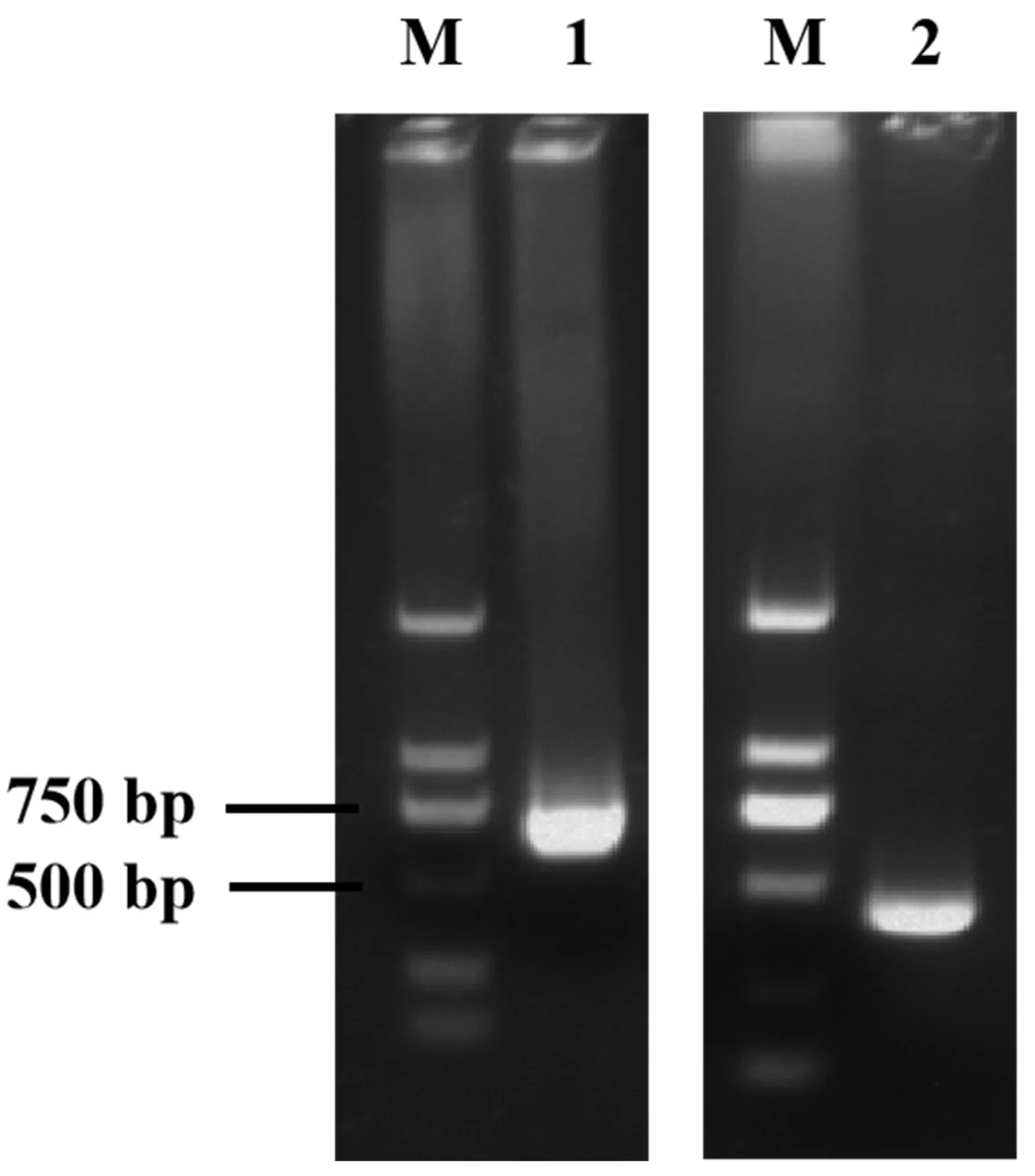
The *HcTGA5* gene RACE Results. (1) *HcTGA5*-*3′* RACE result; (2) *HcTGA5*-*5′* RACE result; M: DNA Marker 2000.

Based on the identified kenaf gene fragments, the designed 3′ end primers were used for amplification via RACE technology, employing kenaf stem and leaf RNA as templates for two PCR reactions, with the resulting products subsequently analyzed using agarose gel electrophoresis. After analyzing the obtained images, a band measuring approximately 684 bp was observed. The primers at the 5′ end were designed using the same method, and a band measuring approximately 412 bp was obtained (Figure 3).

### 2.3. Analysis of Root-Knot Nematode Resistance-Related HcTGA5 Gene

The 5′ RACE and 3′ RACE sequences were spliced using DNAman software (V7.0), resulting in the acquisition of a full-length sequence of the *HcTGA5* gene after excising the vector sequences. DNAstar software (V17.1) was used for the physical and chemical analysis of the full-length sequence. *HcTGA5* gene has a total length of 798 bp and an open reading frame of 693 bp (28–720), which encodes 230 amino acids (accession number: PZ944973). The isoelectric point of HcTGA5 protein is 5.32, and the molecular weight of 26.119 kDa. The base composition is as follows: A = 22.80% [158], T = 22.37% [155], C = 24.96% [173], G = 29.87% [207], undetermined bases = 0.00% [0], (A + T) = 45.17% [313], (C + G) = 54.83% [380].

**Figure 4:**
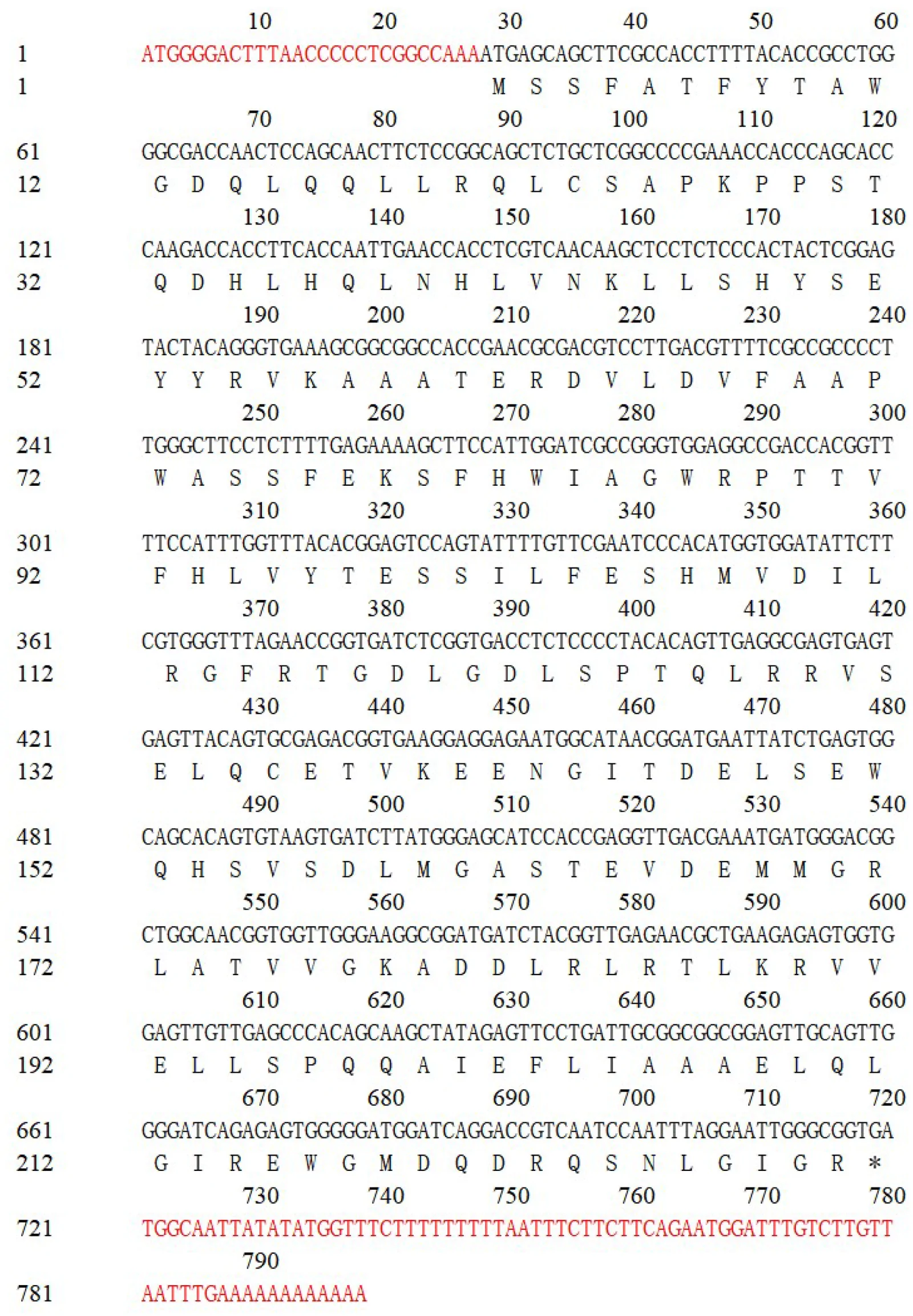
Nucleotide sequence and speculated amino acid sequence of the *HcTGA5* gene. The numbers on the left are nucleotide and amino acid numbers, respectively. Red sequences stand for the 5′-UTR, 3′-UTR and poly(A) tail sequences amplified via RACE-PCR. The asterisk (*) denotes the stop codon for protein translation of *HcTGA5* gene.

The amino acid composition of the product encoded by the *HcTGA5* gene includes 20 basic amino acids, encompassing all types of amino acids. Leucine exhibits the highest repetition frequency (29), followed by serine (22) and glycine (17), while cysteine (2), asparagine (4), and tyrosine (5) demonstrate the lowest repetition frequency. According to the classification of amino acids, there are 110 non-polar amino acids, constituting 47.82% of the total; 59 uncharged polar amino acids, comprising 25.65% of the total; 30 positively charged amino acids, representing 13.04% of the total; and 31 negatively charged amino acids, accounting for 13.48% of the total (Figure 4).

### 2.4. Secondary Structure and Domain of Kenaf HcTGA5 Protein

**Figure 5:**
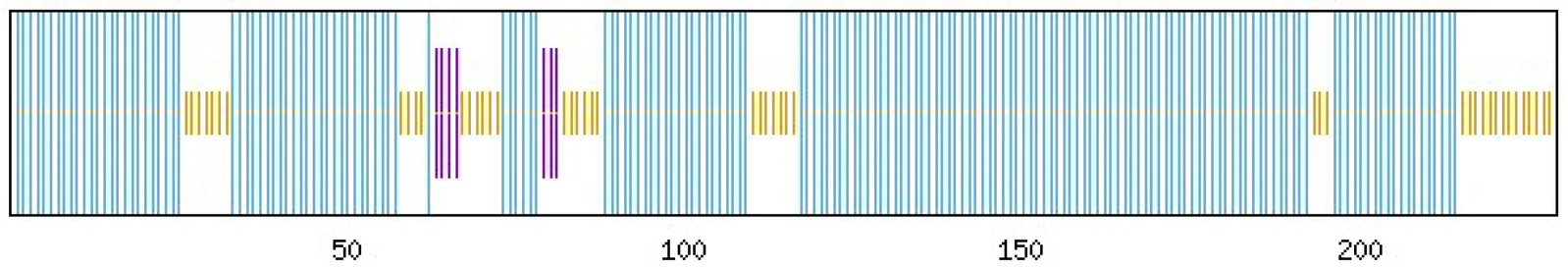
Secondary structure of kenaf HcTGA5 protein.

**Figure 6:**
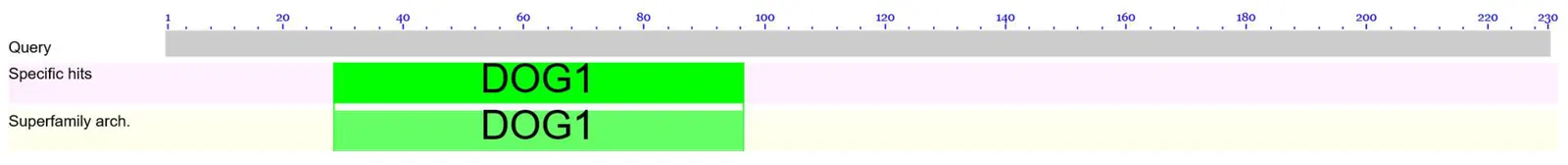
Prediction of conserved region structure of HcTGA5 protein.

The amino acid sequence of the protein encoded by *HcTGA5* gene was analyzed using DNAstar software. The secondary structure analysis of the protein revealed that the α helix and random coil constituted 76.52% and 20.43%, respectively, as the predominant components of the secondary structure, whereas the extension chain comprised 3.04%, respectively (Figure 5). Analysis of the conserved domain of HcTGA5 protein using NCBI showed the presence of DOG1 domain, indicating that kenaf HcTGA5 protein had the conserved domain characteristic of plant TGA5 proteins (Figure 6).

### 2.5. Homology Comparison and Phylogenetic Analysis of Amino Acids Encoded by the Kenaf HcTGA5 Gene

Upon comparing the amino acid sequence of the cloned root-knot nematode resistance-related gene *HcTGA5* in kenaf with the *TGA5* amino acid sequences of other crops obtained from the NCBI database (Figure S2), it was determined that the kenaf HcTGA5 protein and the TGA5 protein of the majority of species possess the DOG1 domain. The DOG1 domain of most plant species contains reasonably conserved regions and features several identical amino acid sequences.

The phylogenetic analysis of the kenaf HcTGA5 protein with other species highlighted that all the protein sequences were approximately divided into five groups (Figure S3). HcTGA5 protein is closely related to disease-resistant proteins of *Olea europaea* subsp. europaea and *Solanum tuberosum*, which are annotated as *TGA5* transcription factors. The second closely related species is *Gossypium australe* and *Quillaja saponaria*, which encode a protein known as DOG1-like 4 and a transcription factor referred to as *TGA5*. Collectively, these phylogenetic results infer that HcTGA5 protein is a *TGA*-like protein and may be significantly involved in plant disease-resistant responses.

### 2.6. Response Pattern of HcTGA5 Gene Related to Root-Knot Nematode Resistance in Kenaf to Hormonal Stimuli

**Figure 7:**
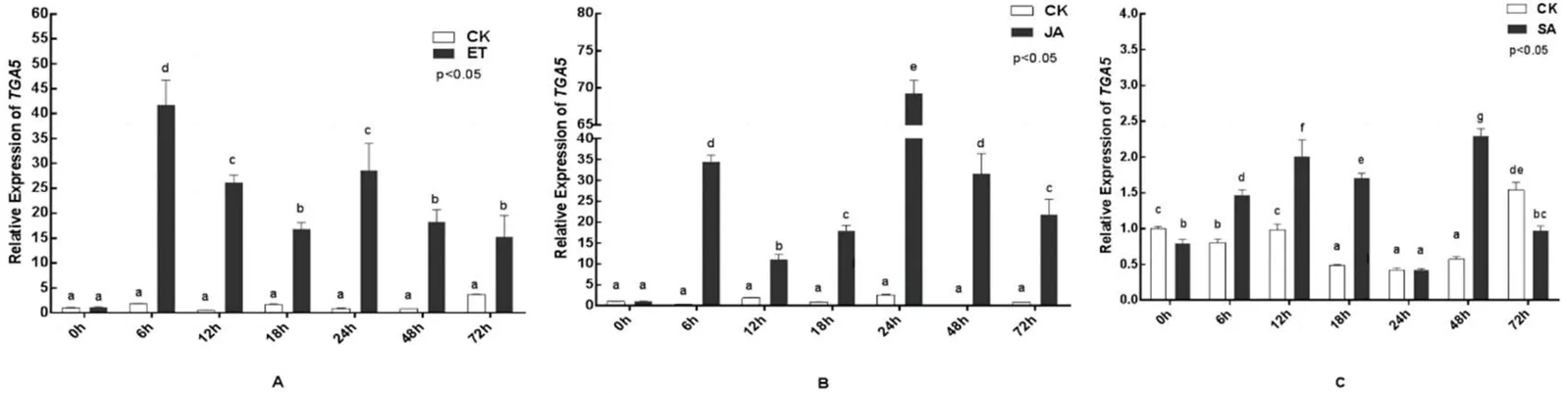
Analysis of expression characteristics of *HcTGA5* gene under different stress treatments. (**A**) Various times after ET treatment; (**B**) Various times after JA treatment; (**C**) Various times after SA treatment; CK: water control treatment. The lowercase letters indicate a statistically significant difference at the level of *p* < 0.05.

Salicylic acid (SA), jasmonic acid (JA), and ethylene (ET) are crucial signaling chemicals in the pathways of plant disease resistance. To evaluate the expression level of the *HcTGA5* gene in kenaf regarding resistance to root-knot nematodes and its role in the nematode-resistant response, RNA was collected from kenaf seedlings subjected to three distinct hormones at multiple time intervals. The isolated RNA was subsequently reverse-transcribed into cDNA. The *HcTGA5* gene’s reaction to the three hormones was evaluated using qRT-PCR. The findings indicated that the expression of the *HcTGA5* gene altered markedly in kenaf following exposure to JA, SA, and ET treatments. The expression pattern of the *HcTGA5* gene following treatment with SA and JA was fundamentally similar. Following ET treatment, the expression of the *HcTGA5* gene peaked at 42 times that of the control at 6 h, subsequently declining. The induction of SA and JA peaked after 48 h (2.3-fold increase compared to control) and 24 h (69-fold increase compared to control), respectively. Furthermore, JA and ET promote *HcTGA5* gene expression more efficiently than SA (Figure 7). All three hormones could stimulate *HcTGA5* gene expression, with the gene exhibiting a very robust response during the initial phase of induction.

### 2.7. The HcTGA5 Gene Response Patterns Following Root-Knot Nematode Infection in Susceptible and Kenaf-Disease Resistant Germplasm

**Figure 8:**
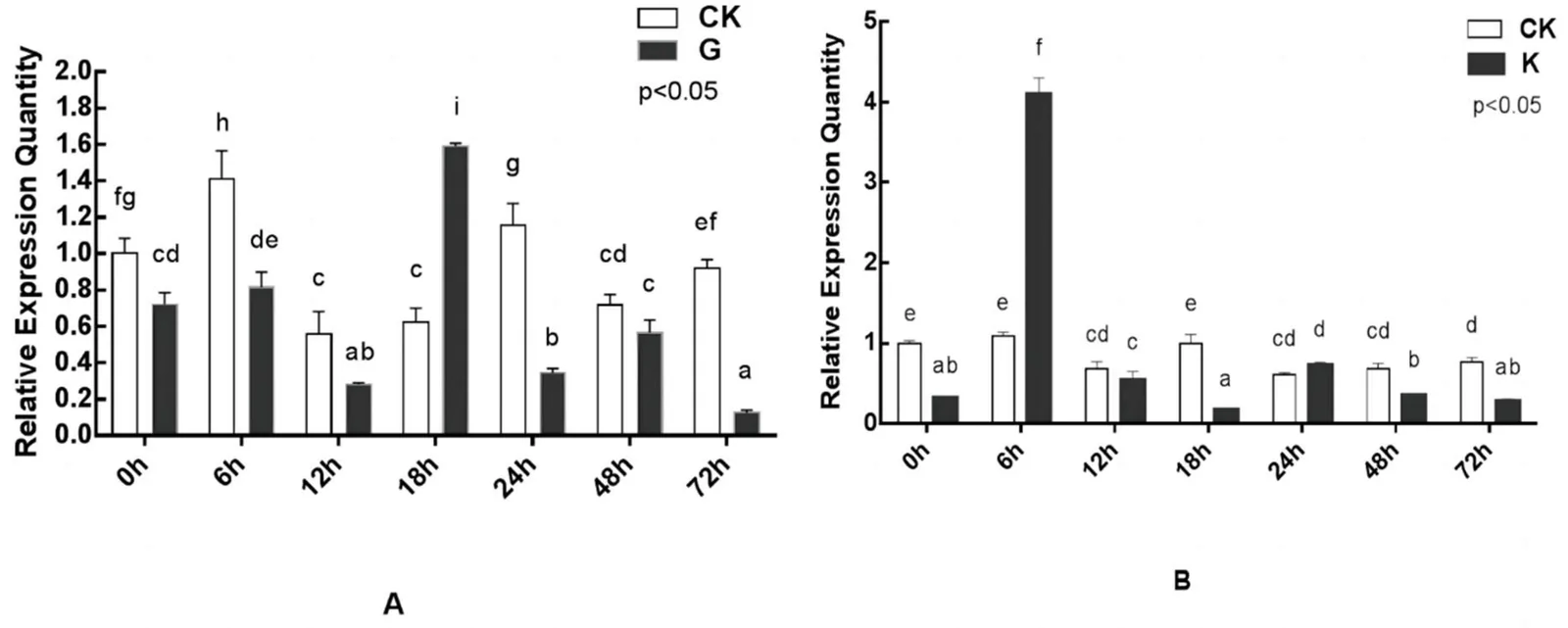
The *HcTGA5* gene is differentially expressed in kenaf-disease-resistant germplasm versus susceptible germplasm. (**A**) kenaf-susceptible cultivar “4391”; (**B**) disease-resistant cultivar “ACC-NO-4114”; G: “4391” inoculated with *M. enterolobii* J2; K: “ACC-NO-4114” inoculated with *M. enterolobii* J2; CK: water control treatment. The lowercase letters indicate a statistically significant difference at the level of *p* < 0.05.

The results showed that the expression of *HcTGA5* gene in the resistant kenaf cultivar “ACC-NO-4114” treated with water (CK) remained relatively unchanged throughout time (Figure 8). However, after inoculation with J2, its expression initially increased and subsequently decreased, peaking at 6 h, which was 4 times higher than the lowest expression level. This indicates that J2 could induce the positive expression of *HcTGA5* gene in kenaf. The expression trend of the susceptible kenaf cultivar “4391” differed from that of the CK group. Following inoculation with J2, its expression exhibited a wavy pattern, while the CK group showed the opposite trend. At 18 h post-root-knot nematode infection, the expression of *HcTGA5* gene was merely 1.6 times that of the control, suggesting that the infection had little effect on the susceptible kenaf cultivar “4391”. *HcTGA5* gene is hypothesized to contribute to kenaf’s resistance to nematodes.

### 2.8. Analysis of the Heat Map of the Expression Patterns of HcTGA5 Gene After Treatment with Meloidogyne enterolobii

**Figure 9:**
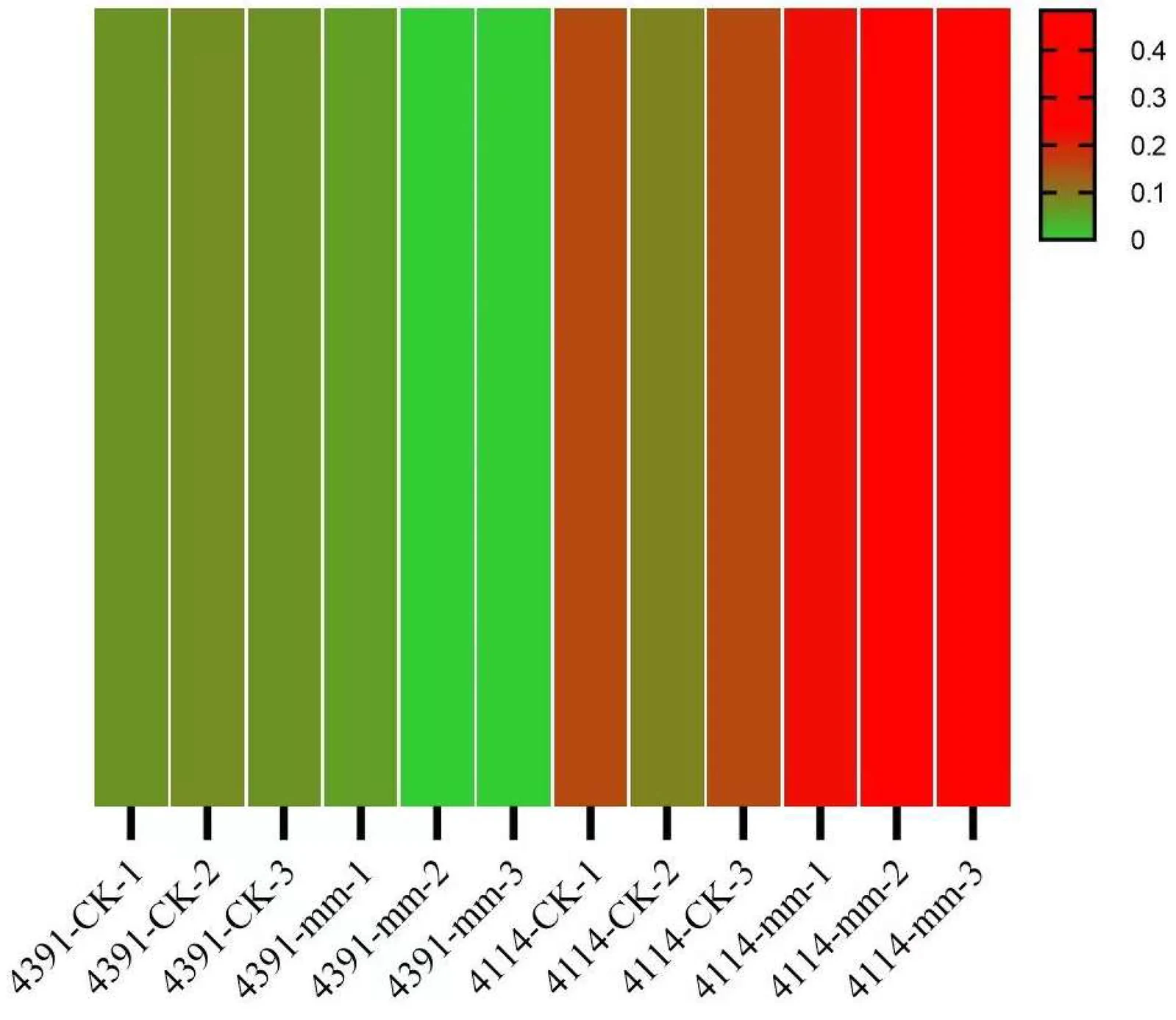
The heatmap shows *HcTGA5* gene expression based on FPKM values in resistant cultivar “ACC-NO-4114” and susceptible cultivar “4391” under *Meloidogyne enterolobii* infection. The abscissa denotes sample names, whereas colors signify gene expression. Red indicates high expression and green signifies low expression.

To further comprehend the expression pattern of *HcTGA5* gene, the expression levels of this gene were analyzed in kenaf varieties with varying resistances after treatment with *M. enterolobii* (Figure 9). Significant variations in *HcTGA5* gene expression were found between the resistant germplasm kenaf variety “ACC-NO-4114” and the susceptible germplasm kenaf variety “4391” during the two-week growth stage following *M. enterolobii* treatment. *HcTGA5* gene expression rose significantly in the resistant germplasm kenaf variety “ACC-NO-4114” following exposure to *M. enterolobii*. In contrast, the susceptible germplasm kenaf variety “4391” exhibited a lack of *HcTGA5* gene expression post-treatment; rather, its expression trended downward. Overall, *HcTGA5* gene expression in the resistant kenaf variety “ACC-NO-4114” was consistently higher than in the susceptible germplasm kenaf variety “4391”, both before and after inoculation. These findings revealed that the regulatory mechanism of *HcTGA5* gene in responding to *M. enterolobii* stress likely played a key role in mediating resistance to this nematode, thereby contributing to the resistant germplasm phenotype of “ACC-NO-4114”.

## 3. Discussion

The TGACG motif-binding factor (*TGA*) gene family is the most significant group within the basic leucine zipper (bZIP) transcription factor family. The *TGA* gene can interact with the activation sequence 1 (AS-1) region on the promoter of the target gene, thereby modulating the transcription levels of downstream target genes and influencing plant resistance or floral organ development [21]. This factor has significant potential for practical applications [22]. In this study, based on the transcriptomic data of kenaf, the full-length *TGA5* gene was cloned using the RACE method and designated *HcTGA5* gene. The HcTGA5 protein has a conserved domain termed DOG1, which is common in most plant TGA proteins. The DOG1 domain in *Arabidopsis thaliana* has been demonstrated to be associated with seed dormancy [23]. The conserved DOG1 region in certain bZIP families may signify the unique gene function of these transcription factors. In addition, DOG1 is the pivotal domain responsible for the disease-resistance-related transcriptional activation of *TGA* transcription factors [24]. The kenaf *HcTGA5* gene function can be anticipated through studying *TGA* genes from other species. Since the identification of the first *TGA* gene in tobacco, *TGA* transcription factors have been isolated in species like rice and apple [25,26,27,28]. The *Arabidopsis thaliana TGA* family contains not only the N-terminal conserved bZIP functional domain but also two glutamine-rich domains (Q1 and Q2) in the C-terminal; additionally, the N-terminal of several members contains conserved STDxDT phosphorylation sites [17]. A series of studies indicates that members of the *TGA1-TGA7* family play a critical role in the disease resistance response of plants [29,30,31]. Meanwhile, truncated *TGA* proteins lacking the complete bZIP domain can interact with other family members and participate in plant disease-resistance regulation via a unique pathway distinct from canonical *TGA* transcription factors. In potato, the *StTGA2.1*-*StTGA2.3* heterodimer module has been verified to be involved in salicylic acid-mediated plant immune responses [32]. Notably, the HcTGA5 protein lacks the complete canonical bZIP domain, which is essential for independent DNA binding in typical *TGA* transcription factors. Nevertheless, consistent with the functional characteristics of reported truncated mini-*TGA* members, *HcTGA5* gene may execute its immune regulatory functions through heterodimerization with intact *TGA* family partners rather than directly binding to downstream promoter elements. However, whether *HcTGA5* gene functions as a transcription factor requires further experimental validation, and its interactions with other TGA protein members remain to be experimentally confirmed.

Plant hormones are essential for the growth and development of plants, as well as for the signal transduction pathways associated with disease and resilience to stress [31]. Zhao et al. (2023) cloned an *ScTGA1* gene, which was constitutively expressed in sugarcane tissues and up-regulated by SA, MeJA, and *Sporisorium scitamineum* stresses [33]. Multiple studies have shown that *TGA* transcription factors participate in disease and stress resistance pathways, including those regulated by SA, JA, ETH, and ABA, hence improving tolerance to biotic and abiotic stimuli [34]. Considering that JA, SA, and ET are important signaling molecules in the plant disease resistance signaling pathway, we analyzed the response patterns of *HcTGA5* gene to JA, SA, and ET. The findings indicated that the expression of the *HcTGA5* gene altered markedly in kenaf following exposure to JA, SA, and ET stress. Following ET treatment, the expression of the *HcTGA5* gene peaked at 42 times that of the control at 6 h, subsequently declining. The induction of SA and JA peaked at 48 h, being 2.3-fold greater than the control, and at 24 h, being 69-fold greater than the control, respectively. Furthermore, JA and ET promote *HcTGA5* gene expression more efficiently than SA. Consequently, it is hypothesized that *HcTGA5* gene contributes to the resistance mechanism of kenaf against nematodes. The findings of this study may provide a basis for subsequent research on kenaf’s resistance to root-knot nematodes. Moreover, it can provide a robust theoretical foundation for the genetic improvement and management of root-knot nematodes in kenaf.

Members of the bZIP transcription factor family play a vital role in the plant hormone-mediated disease and stress resistance signaling pathways. Research indicates that ectopic overexpression of the melon *TGA* gene in *A. thaliana* could improve plant disease resistance [17]. Comprehending the gene function of the *TGA* gene family will establish the foundation for examining the regulatory network of plant hormone-mediated resistance signals. It will additionally determine a framework for the acquisition and application of novel resistance-related genes. In this study, the expression level of the *HcTGA5* gene was substantially boosted in disease-resistant germplasm when infected by pathogens, consistently surpassing the expression level observed in susceptible germplasm. Cloning genes associated with plant disease resistance and studying their expression patterns are essential steps for identifying disease-resistant genes and developing new disease-resistant plant varieties. This study investigated the mechanism by which the *HcTGA5* gene provides resistance to the kenaf root-knot nematode. It utilized a range of kenaf-resistant germplasm resources and employed transcriptome sequencing. The findings demonstrated the gene’s role in conferring resistance, providing a theoretical foundation for the genetic enhancement and management of root-knot nematodes in kenaf.

## 4. Materials and Methods

### 4.1. Plant Materials and Main Reagents

The susceptible kenaf cultivar “4391” and resistant cultivar “ACC-NO-4114” adopted in this experiment were provided by the Institute of Bast Fiber Crops, Chinese Academy of Agricultural Sciences. All experiments were carried out in the nursery of the Institute of Plant Protection at the Hainan Academy of Agricultural Sciences in Haikou, China. The potting media consisted of a mixture of laterite, organic fertilizer, and coconut husk in a 1:1:1 ratio, sterilized at 121 °C for 2 h. The flowerpots used possessed a diameter and height of 20 cm each. Ten kenaf plants were cultivated in each container and irrigated consistently.

The primary reagents utilized in the experiment were as follows: the Trizol extraction kit (No. B511321) and the column DNA gel recovery kit (No. B518131), both obtained from Sangon Biotech. RevertAid Premium Reverse Transcriptase (No. EP0731) was supplied by Thermo Fisher, while LA Taq (No. DRR02AG) and SMARTer RACE 5′/3′ Kit (No. 634858) were provided by TaKaRa. Additionally, SuperReal PreMix Plus SYBR Green (FP171206) was sourced from Tiangen Biotech (Beijing) Co., Ltd.

### 4.2. Experimental Methods

#### 4.2.1. Identification of the HcTGA5 Gene Related to Root-Knot Nematode Resistance in Kenaf

The susceptible kenaf cultivar “4391” and resistant cultivar “ACC-NO-4114” were cultivated in pots with sterile substrates, with eleven plants per germplasm. Once the seedlings demonstrated clear root differentiation, each pot was inoculated with 1000 second-stage juveniles (J2) of *M. enterolobii*, with five pots per germplasm. An additional three pots treated with water alone served as the blank control. The remaining three pots were left untreated. Root tips (approximately 1.5 cm in length) were collected from both uninoculated plants and those inoculated 18 h prior, with three samples taken from each group [35]. These samples were repeatedly frozen in liquid nitrogen and subsequently stored at −80 °C for future analysis. They were then sent to Beijing Biomarker Company for transcriptome sequencing analysis.

High-throughput sequencing was performed to explore the transcriptomic responses of highly resistant and highly susceptible kenaf germplasms upon root-knot nematode infection. After library construction, each paired-end cDNA library was prepared according to the TruSeq RNA Sample Preparation Guide (Illumina, San Diego, CA, USA). The cDNA libraries were sequenced on the HiSeq 2000 platform. Data quality control was performed using fastp to remove reads with adapters. Paired reads were removed under the following conditions: when the number of N in any sequencing read exceeded 10% of the length of that read, and when any sequencing read contained low-quality bases (Q < =20) exceeding 50% of the length of that read. Subsequent analyses were based on clean reads. The reference genome (GWHACDB00000000.genome.fasta.gz) and its annotation files (GWHACDB00000000.gff.gz) were downloaded from a specified website (https://download.cncb.ac.cn/gwh/Plants/Hibiscus_cannabinus_Kenaf_genome_of_Fuhong952_GWHACDB00000000/) (accessed on 10 January 2026). HISAT2 was used to build an index, and clean reads were aligned to the reference genome. Gene expression levels were quantified using featureCounts to calculate gene alignment statistics. Subsequently, FPKM (Fragments Per Kilobase Million) values for each gene were computed based on gene length. DESeq2 was used for differential gene expression analysis between two groups. Enrichment analysis was performed based on the hypergeometric test, with pathway-based hypergeometric distribution testing for KEGG and GO term-based analysis for GO.

#### 4.2.2. Total RNA Isolation and Synthesis of First-Strand cDNA

Root tissues were collected from the resistant kenaf cultivar “ACC-NO-4114”. The processes to acquire kenaf total RNA using Sangon Biotech’s Trizol extraction kit (No. B511321) were carried out precisely as prescribed. DNase I was used to eliminate residual genomic DNA before subsequent experiments. RNA purity was detected by spectrophotometry, and RNA integrity was identified using agarose gel electrophoresis. Each analysis was performed independently three times.

A total of 5 µL of RNA, 1 µL of random primer, and 1 µL of ddH_2_O were combined in a 0.2 mL PCR tube. The mixture was heated to 70 °C for 5 min and then placed in an ice bath for 2 min. Following this, the reagents were added via centrifugation: 2 µL of 5X First-Strand Buffer, 0.5 µL of a 10 mmol dNTP solution, 0.25 µL of RNase inhibitor, and 0.25 µL of Reverse Transcriptase, resulting in a total volume of 10 µL. The resulting mixture was incubated at 42 °C for 60 min, followed by a final incubation at 72 °C for 10 min.

#### 4.2.3. 3′ RACE cDNA Sequencing of HcTGA5 Gene in Kenaf

The 3′-RACE experiment was performed using the SMARTer RACE 5′/3′ Kit Kit (TaKaRa). First-strand cDNA was synthesized by reverse transcription from total RNA using the kit components following the manufacturer’s instructions. Nested amplification was then conducted in two successive rounds. In the first round, the reaction system included: 25 µL of 2×Phanta Max Buffer, 2 µL of 10 µM *TGA5*-3GSP1, 2 µL of 10 µM 5.3′ outer primer, 1 µL of 10 mM dNTP Mix, 2 µL of cDNA, 1 µL of 1 U/µL Phanta Max Super-Fidelity DNA Polymease, and 17 µL of ddH_2_O. The procedure was as follows: pre-denaturation at 95 °C for 3 min, followed by 25 cycles consisting of denaturation at 94 °C for 30 s, annealing at 58 °C for 30 s, and extension at 72 °C for 2 min, concluding with a final extension at 72 °C for 5 min.

In the second round, the reaction system contained: 25 µL of 2×Phanta Max Buffer, 2 µL of 10 µM *TGA5*-3GSP2, 2 µL of 10 µM 5.3′ inner primer, 1 µL of 10 mM dNTP Mix, 2 µL of the diluted product from the first-round PCR, 1 µL of 1 U/µL Phanta Max Super-Fidelity DNA Polymease, and 17 µL of ddH_2_O. The procedure involved pre-denaturation at 95 °C for 3 min, followed by 30 cycles of denaturation at 94 °C for 30 s, annealing at 58 °C for 30 s, and extension at 72 °C for 2 min, finishing with a final extension at 72 °C for 5 min. The relevant primer sequences are listed in Table S1.

PCR products were purified by gel extraction, ligated into the pMD18-T vector, and subsequently transformed into competent cells (SK2301) using a heat-shock method. Positive clones were selected and sequenced by Sangon Biotech, ShangHai, China.

#### 4.2.4. Amplification of 5′ RACE of HcTGA5 Gene cDNA Sequence

The 3′-RACE experiment was performed using the SMARTer RACE 5′/3′ Kit Kit (TaKaRa). First-strand cDNA was synthesized by reverse transcription from total RNA using the kit components following the manufacturer’s instructions. Nested amplification was then conducted in two successive rounds. In the first round, the reaction system included: 25 µL of 2×Phanta Max Buffer, 2 µL of 10 µM *TGA5*-5GSP1, 2 µL of 10 µM Universal Primer, 1 µL of 10 mM dNTP Mix, 2 µL of cDNA, 1 µL of 1 U/µL Phanta Max Super-Fidelity DNA Polymease, and 17 μL of ddH_2_O. The procedure was as follows: pre-denaturation at 95 °C for 3 min, followed by 25 cycles consisting of denaturation at 94 °C for 30 s, annealing at 58 °C for 30 s, and extension at 72 °C for 2 min, culminating in a final extension at 72 °C for 5 min.

For the second round, the reaction system contained: 25 µL of 2×Phanta Max Buffer, 2 µL of 10 µM *TGA5*-5GSP2, 2 µL of 10 µM Universal Short, 1 µL of 10 mM dNTP Mix, 2 µL of diluted product from the first round PCR, 1 µL of 1 U/µL Phanta Max Super-Fidelity DNA Polymease, and 17 µL of ddH_2_O. The procedure involved pre-denaturation at 95 °C for 3 min, followed by 30 cycles of denaturation at 94 °C for 30 s, annealing at 60 °C for 30 s, and extension at 72 °C for 2 min, concluding with a final extension at 72 °C for 5 min. The relevant primer sequences are detailed in Table S1. PCR products were purified using gel extraction as explained earlier.

#### 4.2.5. Full-Length Splicing and Sequence Analysis of HcTGA5 Gene

The amplified 5′ RACE and 3′ RACE sequences were spliced using DNAman software, and the vector sequence was removed to yield the full-length sequence *HcTGA5* gene. DNAstar software was employed for the physicochemical property analysis of the full-length sequence. MegAlign software (V17.1) was used to assess the homology of nucleotides and their corresponding encoded amino acids. Additionally, MEGA software (V11.0) was employed to assess amino acid homology and generate a phylogenetic tree.

#### 4.2.6. Expression Profile of HcTGA5 Gene

When susceptible kenaf cultivar “4391” developed 4 to 5 true leaves, uniform seedlings were transferred into individual culture flasks and root-soaked separately in 200 mL solutions of 1 mmol/L jasmonic acid (JA), 1 mmol/L salicylic acid (SA), and 2 mmol/L ethephon. Seedling tissues were harvested at 0 h (immediately), 6 h, 12 h, 18 h, 24 h, 48 h and 72 h post-treatment. All collected samples were snap-frozen in liquid nitrogen and stored at −80 °C for subsequent molecular assays. Seedlings immersed in an equal volume of solvent-matched sterile distilled water were set as the blank control. Three independent biological replicates were arranged for every treatment and time point. Total RNA samples underwent reverse transcription analysis using the following procedure: incubation at 42 °C for 30 min, and the reaction was terminated by heating at 85 °C for 5 s. A 20 µL reverse transcription system containing 2 µg of total RNA was then cooled to subsequently stored at 4 °C. Fluorescence quantification was carried out using a CFX-96 Touch instrument (BIO-RAD). For the subsequent PCR step, the reverse-transcribed cDNA served as the template, with a total reaction volume of 20 µL. The system included 2 µL of cDNA template, 0.7 µL of each upstream and downstream primer (sequences provided in Table S1), 10 µL of 2X Super RealpreMixplus, and 6.6 µL of RNase-free ddH2O. Each treatment was performed in triplicate, with the following reaction conditions: pre-denaturation at 95 °C for 5 min, followed by 40 cycles consisting of denaturation at 95 °C for 10 s, annealing at 60 °C for 20 s, and extension at 72 °C for 20 s. A melting curve was generated from 65 °C to 95 °C, and CT values were recorded at each temperature point. Three technical replicates were arranged for each qRT-PCR reaction. The relative expression levels of *HcTGA5* gene were quantified using the 2^−△△CT^ method [36]. Meanwhile, the kenaf *HcACT7* gene was adopted as the internal reference gene for kenaf tissue detection [37].

#### 4.2.7. Application of Root-Knot Nematode Infection

Kenaf plants from two germplasm types were used in the study: a disease-resistant cultivar “ACC-NO-4114” and a susceptible cultivar “4391”. Upon reaching the 3–4 leaf stage, each seedling of “ACC-NO-4114” (marked as K) and “4391” (marked as G) was inoculated with 1000 J2 nematodes. Untreated kenaf plants, which were watered but not inoculated, served as blank controls (marked as CK). Root samples were collected at various time intervals, including 0 h, 6 h, 12 h, 18 h, 24 h, 48 h, and 72 h post-treatment—immediately frozen in liquid nitrogen, and stored at −80 °C. Each treatment consisted of four biological replicates. The samples were then analyzed by qRT-PCR to assess the differential expression of the *HcTGA5* gene between the two germplasms, and the experimental procedures were performed in accordance with Section 4.2.6.

#### 4.2.8. Analysis of the Heat Map of the Expression Pattern of HcTGA5 Gene

Two plant varieties were studied: one demonstrating high resistance cultivar “ACC-NO-4114” and the other showing high susceptibility cultivar “4391”. These were assigned to two groups: a control group (CK) treated with water and a treatment group (mm) exposed to 1000 J2 of *M. enterolobii*. In total, there were four sample groups. Each group had three biological replicates, and samples were collected for transcriptome sequencing two weeks after the inoculation with *M. enterolobii*. The FPKM values of *HcTGA5* gene, which indicate the expression levels of *HcTGA5* gene under *M. enterolobii* treatment, were extracted. A heat map was then generated to analyze the differential gene expression patterns.

#### 4.2.9. Statistical Analysis

Data statistics were performed using Excel, one-way analysis of variance (ANOVA) was performed using IBM SPSS Statistics 25, and statistical significance was defined as *p* < 0.05. Data visualization was conducted using Origin 2018.

## 5. Conclusions

In this study, the *HcTGA5* gene was first identified from kenaf transcriptome sequencing data, followed by successful cloning of its full-length cDNA. Three phytohormones, including jasmonic acid (JA), salicylic acid (SA), and ethylene (ET), were all shown to induce the expression of *HcTGA5* gene in kenaf, with its expression peaking at 24 h, 48 h, and 6 h post-treatment for JA, SA, and ET, respectively. Furthermore, comparative analysis revealed significantly higher *HcTGA5* gene expression in nematode-resistant kenaf germplasms than in susceptible counterparts, directly supporting its potential role in mediating nematode resistance. Further studies are required to elucidate the specific signaling pathways through which *HcTGA5* gene modulates kenaf’s defense responses to *M. enterolobii*. Nevertheless, this study lays a solid theoretical foundation for further clarifying kenaf’s nematode resistance mechanisms, while also providing valuable genetic resources for kenaf genetic improvement and the management of root-knot nematodes in agricultural practices.

## Data Availability

All data generated and analyzed during this study are fully included within the article. The corresponding author will provide the original datasets upon reasonable request from readers or journal staff.

## Supplementary Materials

The following supporting information can be downloaded at: https://www.mdpi.com/article/10.3390/ijms27188249/s1.

## Abbreviations

*M. enterolobii*	*Meloidogyne enterolobii*
qRT-PCR	Quantitative real time reverse transcription-polymerase chain reaction
RT-PCR	Reverse transcription-polymerase chain reaction
JA	Jasmonic acid
SA	Salicylic acid
ET	Ethylene
H	Hour
Min	Minute
CaMV	Cauliflower mosaic virus
GUS	β-glucuronidase
EMSA	Electrophoresis mobility shift assay
RACE	Rapid-amplification of cDNA ends
J2	Second-stage juveniles
NCBI	National Center for Biotechnology Information
