# Supplementary material for: Expression Profiling and Characterization of HcTGA5 Gene in Kenaf Under Hormonal Treatments and Its Association with Resistance to Root-Knot Nematode
Source: Int J Mol Sci. 2026 Sep 16;27(18):8249. doi: 10.3390/ijms27188249 (PMC13607479; doi:10.3390/ijms27188249)
Supplement: Supplementary file 1 [file ijms-27-08249-s001.zip › ijms-4445879-supplementary.pdf]

Supplementary Materials

Table S1. Primer Sequences Used in the Experiment.

Primer Names	Sequence	Purpose
TGA5-3GSP1	CCCTTGGGCTTCCTCTTTTGA	3' RACE specific primer
Outer Primer	TACCGTCGTTCCACTAGTGATTT	
TGA5-3GSP2	ACCTCTCCCCTACACAGTTGA	
Inner Primer	CGCGGATCCTCCACTAGTGATTTCACT ATAGG	
TGA5-5GSP1	AACTCCGCCGCCGCAATCAGGAACTC	5' RACE specific primer
Universal Primer	TAATACGACTCACTATAGGGCAAGCA GTGGTATCAACGCAGAGT	
TGA5-5GSP2	TCCTTCACCGTCTCGCACTGTAAC	
Universal Short	GTATCAACGCAGAGTACGG	
T-f4	ACAGTGTAAGTGATCTTATGGG	*TGA5* qPCR target gene primer
T-r4	CGCCGCAATCAGGAACTCTA	*TGA5* qPCR target gene primer
HcACT7-F	GATATTCAACCCCTTGTCTGTG	qPCR internal reference primer
HcACT7-R	CTTCTGACCCATCCCAACCAT	qPCR internal reference primer

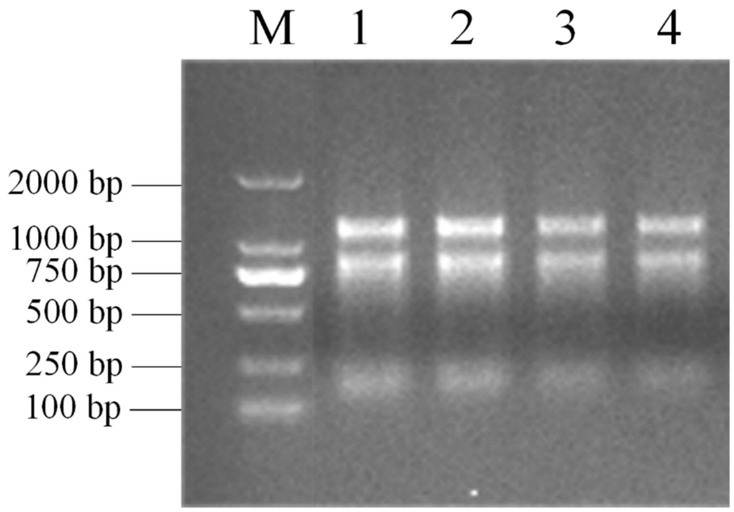

Figure S1. Electrophoresis identification results of kenaf RNA.

```
1__*Kenaf_HcTGA5       ...........MSSFATFYTA.WGDQLQQLLRQLCSAPK.     27
2__CAA3006644.1        ............MSFQRFHDT.WFEQLRHLVHQLSQAPR.     26
3__KAA3482610.1        .......MASDDQILQRCFHE.WMAIQEQELNQLIQALNQ     32
4__KAJ7957071.1        MSNITSSDNSERESFQQFFES.WLVQQNRDLQDLIFASTN     39
5__KAK1261958.1        ............MSFESFFQG.WLVRQEELLDELLSAPR.     26
6__KAK1286341.1        ............MSFESFFQG.WLDRQEELLDELLSAPR.     26
7__OIT24592.1          .......MATAYLKSQRFISSGWLIQQEFFLQQLLRLTC.     32
8__XP__006348396.1     ............MSFQRFYDT.WFDQLKEIVRQLSQVPR.     26
Consensus                                    w          l

1__*Kenaf_HcTGA5       ...................PPSTQDHLHQLNHLVNKLLSH     48
2__CAA3006644.1        ...................PPTTEEDYHHLHQLVNKVMSH     47
3__KAA3482610.1        .................NENGGDDLTETSCAQLTEKSINS     55
4__KAJ7957071.1        HKNTNTNHSKTEATPIADGQVSQMANGKVSKQLINQVIEH     79
5__KAK1261958.1        ....................EGEE...PKLRELIEKALTL     43
6__KAK1286341.1        ....................EGEE...HKLRELIEKALTH     43
7__OIT24592.1          ....................ESNDNCINEERLIIDQVLAH     52
8__XP__006348396.1     ...................PATSEQHRELHQQLVQKVVSH     47
Consensus

1__*Kenaf_HcTGA5       YSEYYRVKAAATERDVLDVFAAPWASSFEKSFHWIAGWRP     88
2__CAA3006644.1        CAEYYRVKSLAIKQDVLSFSTAQWTTTLERSLHWIGGWRP     87
3__KAA3482610.1        FQEYIDKRAQLSRLDISGLFSPSWNTALENSLLWVAGCRP     95
4__KAJ7957071.1        YEHYYKTKSRCVKQDVLAMLSPTWTTSLEDAFLWIGGWRP    119
5__KAK1261958.1        YGAYYREKSLMASRDVLLVFSPRWFTSCERTFLWIAGWKP     83
6__KAK1286341.1        YGAYYREKSLMASRDVLLVFSPRWFTACERTFLWIAGWKP     83
7__OIT24592.1          YCAYYVAKSKVTQENVFLVLSPTWFTPLERTYLWIAGFRP     92
8__XP__006348396.1     IYEYYRVKSLAAKNDILSVYSAPWSTSLERSLHWIAGWRP     87
Consensus                 y                   w    e    w  g  p

1__*Kenaf_HcTGA5       TTVFHLVYTESSILFESHMVDILRGFRTGDLGDLSPTQLR    128
2__CAA3006644.1        TTVFHLVYTESSILFESRVVDILRGFRTGDLGDLSPGQFR    127
3__KAA3482610.1        SMYIRLTYALCGSQVEFQLSEIIQRLARGNLGEISAAQLR    135
4__KAJ7957071.1        SMAFHLLYSKSGLEFEVRFNEIIHGLHSFDLADLSLNQIT    159
5__KAK1261958.1        GMAFRLVRSNVEGLTDEQSEVIERLREGTAAREEELAAEM    123
6__KAK1286341.1        GMAFRLVRSNVEGLTDEQSEAIGRLREGTAEREEELAAEM    123
7__OIT24592.1          GLAFRLVNKNVLDLSRNQSLMLNELLEEIKVEEKQLTDEF    132
8__XP__006348396.1     TTAFHLIYTESSILFESHIIDILRGLRYGDLGDLSPDQLA    127
Consensus                   l

1__*Kenaf_HcTGA5       RVSELQCETVKEENGITDELSEWQHSVSDLMGAST.....    163
2__CAA3006644.1        RVSELQCETVHEENAITDELSEWQDSASDLVGLCG.....    162
3__KAA3482610.1        MINDLHMKTIKEEEKLSNKLAGLQENIADQPIAIVAKRMS    175
4__KAJ7957071.1        KLDELQTKTIREEREITEKMAKQQETVADSSMVELSHLMT    199
5__KAK1261958.1        TMVQEASVLGINLGPRYKH.....................    142
6__KAK1286341.1        TMVQEASVLGINLGPRRWRRNGFALRDGEAVETAAE....    159
7__OIT24592.1          TRVQQTMALETMILLLREE....GLLENGRLTEPNR....    164
8__XP__006348396.1     RVSELQCEAVQEENSITDEFNDWQDSASEILGLMG.....    162
Consensus

1__*Kenaf_HcTGA5       ......................EVDEMMGRLATVVGKADD    181
2__CAA3006644.1        ......................DFDEKMERLARILNKADS    180
3__KAA3482610.1        RVGETSG...........EVDHALDEHESSMANILQEADK    204
4__KAJ7957071.1        EMIRDAGTGRNDSNGFEDRVDSTLAPKEDELEKILLRADD    239
5__KAK1261958.1        ........................................    142
6__KAK1286341.1        ......................AMEVVTGRMESLLSLADS    177
7__OIT24592.1          ......................AIDRLRLAMETLVECADY    182
8__XP__006348396.1     ......................DIEGKMEKLVEILEKADK    180
Consensus

1__*Kenaf_HcTGA5       LRLRTLKRVVELLSPQQAIEFLIAAAELQLGIREWGMDQD    221
2__CAA3006644.1        LRLKTIETVVELLTPQQAAEFLVAAAELHFGIHAWGVEQD    220
3__KAA3482610.1        LRLSTLKELLSILTPLQGVDFLVASKKLHLFMHEWGKTRD    244
4__KAJ7957071.1        LRLRTLKSILNILNPLQAAQFLIAAAELHLRLHDWGKKRD    279
5__KAK1261958.1        ........................................    142
6__KAK1286341.1        LRKKTVMDLVGVLEPLQAIDLFAAATQLHIHLRRWGLQRE    217
7__OIT24592.1          LRGKTVLRIVDILSATQTVRFLAAATQLHLRIRSLALQSE    222
8__XP__006348396.1     LRLKTIENLVELLTPQQAIEFLIASAHLQFGIRRWGINHD    220
Consensus

1__*Kenaf_HcTGA5       RQSNLGIGR...............................    230
2__CAA3006644.1        RQRGNA..................................    226
3__KAA3482610.1        NRHGLLRAQQEGYWFLFYAYSDIISAVPAIRVDAQSVHNP    284
4__KAJ7957071.1        ARYDNRGISSLSNGGHATVDQYGECNS.............    306
5__KAK1261958.1        ........................................    142
6__KAK1286341.1        SGTA....................................    221
7__OIT24592.1          AN......................................    224
8__XP__006348396.1     RQRENP..................................    226
Consensus

1__*Kenaf_HcTGA5       .......                                     230
2__CAA3006644.1        .......                                     226
3__KAA3482610.1        VISLYTC                                     291
4__KAJ7957071.1        .......                                     306
5__KAK1261958.1        .......                                     142
6__KAK1286341.1        .......                                     221
7__OIT24592.1          .......                                     224
8__XP__006348396.1     .......                                     226
Consensus
```

Figure S2. Homology comparison of deduced amino acid sequence of kenaf *HcTGA5* gene with other plant *TGA5* gene. 1 *Kenaf *HcTGA5*; 2 *Olea europaea* subsp. europaea (CAA3006644.1); 3 *Gossypium australe* (KAA3482610.1); 4 *Quillaja saponaria* (KAJ7957071.1); 5 *Acorus gramineus* (KAK1261958.1); 6 *Acorus calamus* (KAK1286341.1) ; 7 *Nicotiana attenuata* (OIT24592.1); 8 *Solanum tuberosum* (XP_006348396.1).

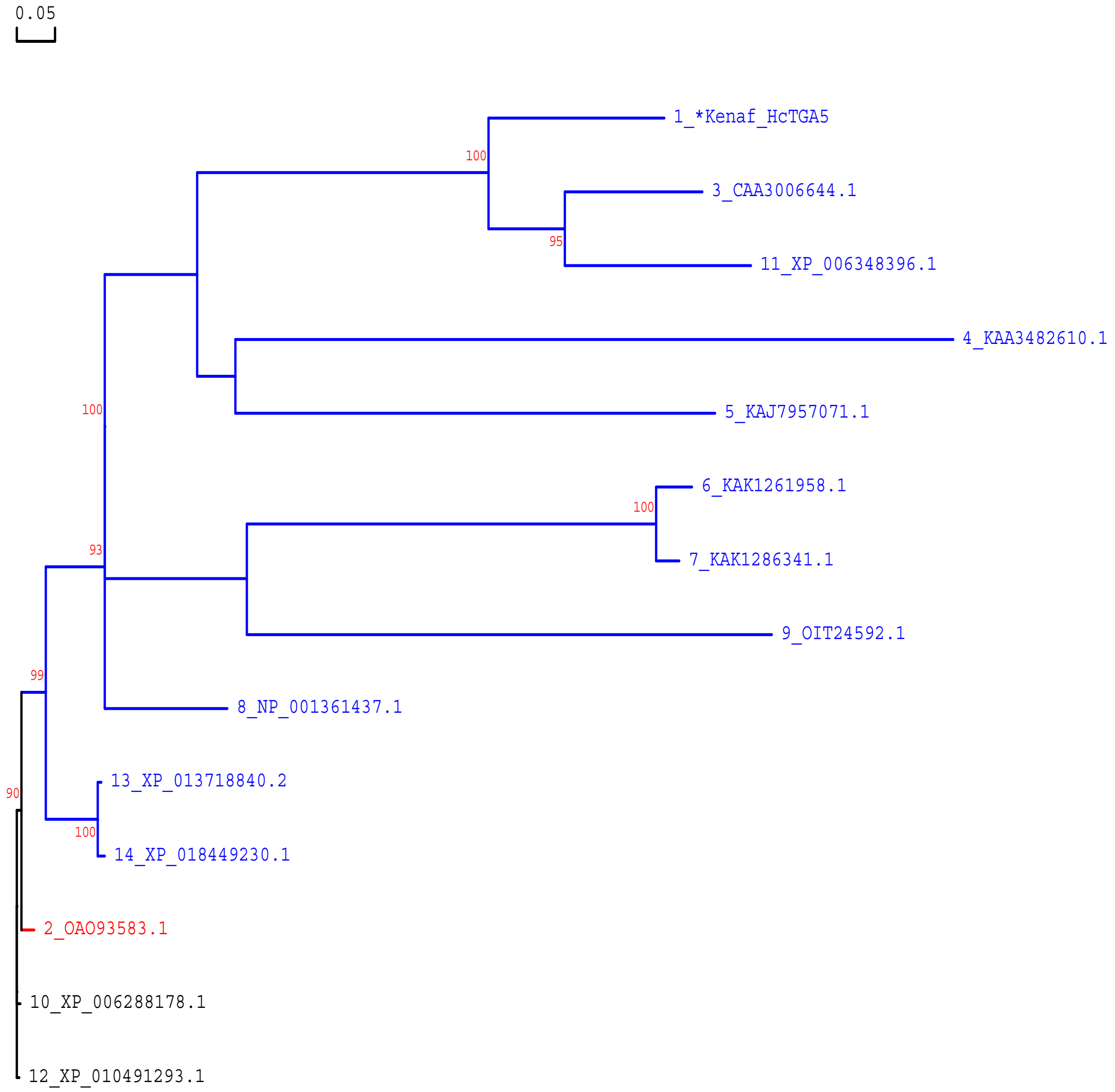

Figure S3. Phylogenetic tree of kenaf *HcTGA5* protein and other plant *TGA5* protein. 1 *Kenaf *HcTGA5*; 2 *Arabidopsis thaliana* (OAO93583.1); 3 *Olea europaea* subsp. europaea (CAA3006644.1); 4 *Gossypium australe* (KAA3482610.1); 5 *Quillaja saponaria* (KAJ7957071.1); 6 *Acorus gramineus* (KAK1261958.1); 7 *Acorus calamus* (KAK1286341.1); 8 *Solanum lycopersicum* (NP_001361437.1); 9 *Nicotiana attenuata* (OIT24592.1); 10 *Capsella rubella* (XP_006288178.1); 11 *Solanum tuberosum* (XP_006348396.1); 12 *Camelina sativa* (XP_010491293.1); 13 *Brassica napus* (XP_013718840.2); 14 *Raphanus sativus* (XP_018449230.1).
